# Krill oil treatment ameliorates lipid metabolism imbalance in chronic unpredicted mild stress-induced depression-like behavior in mice

**DOI:** 10.3389/fcell.2023.1180483

**Published:** 2023-07-26

**Authors:** Hao Zhang, Xiaofang Liu, Bo Li, Yi Zhang, Hua Gao, Xianyong Zhao, Kailiang Leng, Zhenhua Song

**Affiliations:** ^1^ Department of Pharmacology, Qingdao University School of Pharmacy, Qingdao, China; ^2^ Yellow Sea Fisheries Research Institute, Chinese Academy of Fishery Sciences, Qingdao, China

**Keywords:** major depressive disorder, CUMS, krill oil, transcriptome sequencing, lipid metabolism

## Abstract

The pathology of depression involves various factors including the interaction between genes and the environment. The deficiency of n-3 polyunsaturated fatty acids (n-3 PUFAs) in the brain and depressive symptoms are closely related. Krill oil contains abundant amounts of n-3 PUFAs incorporated in phosphatidylcholine. However, the effect of krill oil treatment on depression-like behaviors induced by chronic stress and its molecular mechanism in the brain remain poorly understood. Here, we used a chronic unpredictable mild stress (CUMS) model to evaluate the effect of krill oil on depression-like behaviors and explored its molecular mechanism through lipid metabolomics and mRNA profiles in the whole brain. We observed that CUMS-induced depression-like behaviors were ameliorated by krill oil supplementation in mice. The metabolism of glycerophospholipids and sphingolipids was disrupted by CUMS treatment, which were ameliorated after krill oil supplementation. Further analysis found that differently expressed genes after krill oil supplementation were mainly enriched in the membrane structures and neuroactive ligand–receptor interaction pathway, which may be responsible for the amelioration of CUMS-induced depression-like behaviors. Altogether, our results uncovered the relationship between lipid metabolism and CUMS, and provided new strategies for the prevention and treatment of depression.

## Introduction

Major depressive disorder (MDD) is one of the most common mental disorders with a high prevalence and mortality rate, and is characterized by depressed mood, loss of interest or pleasure in daily activities, and negative cognition after negative emotional stimuli (Kessler and Bromet, 2013; GBD, 2017 Disease and Injury Incidence and Prevalence Collaborators, 2018). According to the statistics of the World Health Organization (WHO), MDD is the world’s fourth leading cause of disability and is expected to be the leading contributor to suicidal deaths as the increasing number of people living with MDD (Murray and Lopez, 1996; Friedrich, 2017). Despite various hypotheses about MDD pathogenesis proposed in the past few years, the pathophysiological mechanisms that underlie MDD remain unclear (Massart et al., 2012; Cai et al., 2015). The application of antidepressants for different mechanisms is the main method to treat depression, but it is ineffective for a large portion of patients and has side effects (Martinowich et al., 2013; Hodes et al., 2015).

Numerous factors, including genetic and environmental factors, can contribute to depression (Wong et al., 2012; Pigoni et al., 2018). Fatty acids, as important nutrients in the diet, are reported to be associated with depression. Multiple follow-up and experimental studies have confirmed that the intake of saturated fatty acids is proportional to the symptoms of depression. Increasing the intake of monounsaturated fatty acids can reduce the risk of depression in women (Wolfe et al., 2009). In epidemiology, ecological studies show that there is a strong negative correlation between the prevalence of depression and fish consumption among populations. People who rarely eat fish have higher rates of depression (Hibbeln, 1998; Chrysohoou et al., 2010; Smith et al., 2014). Moreover, this preventive effect is largely due to the n-3 polyunsaturated fatty acids (n-3 PUFAs) in fish. Compared to n-6 PUFAs, n-3 PUFAs intake was negatively correlated with the incidence of depression (Levant, 2013; Thesing et al., 2018). Peripheral levels of n-3 PUFAs in patients with MDD were lower than those in patients without MDD, and a fish-rich diet was associated with a reduced incidence of depression (Fehily et al., 1981; Lin et al., 2010; Su et al., 2013; Wani et al., 2015). Compared with the placebo, the incident rates of IFN-α-induced depression were significantly lower in EPA-treated patients but not in DHA-treated patients (Su et al., 2014). Neither EPA-enriched nor DHA-enriched n-3 PUFAs were superior to placebo for the treatment of MDD (Mischoulon et al., 2015). In addition, EPA shows greater efficacy as an adjunctive treatment for mild-to-moderate depression than DHA or placebo. Clinical studies have reported that EPA has antidepressant effects, especially when used in combination with DHA (Nemets et al., 2006; Mocking et al., 2016). Although some therapeutic clinical trials have some limitations, they consistently suggest that n-3 PUFAs may have antidepressant effects (Maki et al., 2009; Konagai et al., 2013).

Krill oil is extracted from Antarctic krill and is also a good source of n-3 PUFAs (Gigliotti et al., 2011). Different from fish oil, which contains abundant amounts of n-3 PUFAs stored as triglycerides, krill oil contains a high amount of n-3 PUFAs incorporated in phosphatidylcholine and a lower content of saturated fatty acids (Ulven and Holven, 2015; Ahn et al., 2018; Kim et al., 2020). Many studies provide evidence that krill oil is absorbed more easily through the gastrointestinal wall and that phospholipids–DHA and partial acylglycerols–DHA are efficient carriers of dietary DHA when compared to conventional triacylglycerol–DHA (Ulven and Holven, 2015; Yurko-Mauro et al., 2015; Destaillats et al., 2018). Supplementation of krill oil increased plasma EPA and DHA concentrations (Maki et al., 2009), and krill oil activated cognitive function in elderly subjects by near-infrared spectroscopy and electroencephalography (Konagai et al., 2013). Wibrand et al. (2013) demonstrated that krill oil was effective for improving depression-like behavior in mice (Strekalova et al., 2011; Hill et al., 2012). However, the molecular mechanism by which krill oil treatment ameliorates depression-like behaviors has not been clarified in mice.

In this study, chronic unpredictable mild stress (CUMS), a well-accepted method used to mimic clinical depression, was used to evaluate the effect of krill oil supplementation on depression-like behaviors. The lipid metabolome was analyzed by high-performance liquid chromatography–mass spectrometry (LC-MS), and the mRNA profiles of the whole brain were analyzed by high-throughput sequencing to reveal the molecular changes. This work will provide a solid basis for the understanding of the effect of krill oil supplementation on CUMS-induced depression-like behavior, reveal lipid metabolism in chronic stress, and provide new strategies for the prevention and treatment of MDD.

## Materials and methods

### Mice

For this study, 8-week-old C57BL/6J male mice were purchased from Beijing Vital River Laboratory Animal Technology Co., Ltd. In separate cages, all groups of mice have no restrictions on access to food or water. The mice were housed in a cage of 32 cm × 16 cm × 16 cm with light in the environment between 07:00 and 19:00. The mice were raised in an environment of 22°C ± 2°C and a relative humidity of 55% ± 5%. These standards remain unchanged in CUMS. All experiments were approved by the Animal Use and Care Committee of Qingdao University.

### Procedures for chronic unpredicted mild stress (CUMS) and krill oil supplementation

**FIGURE 1:**
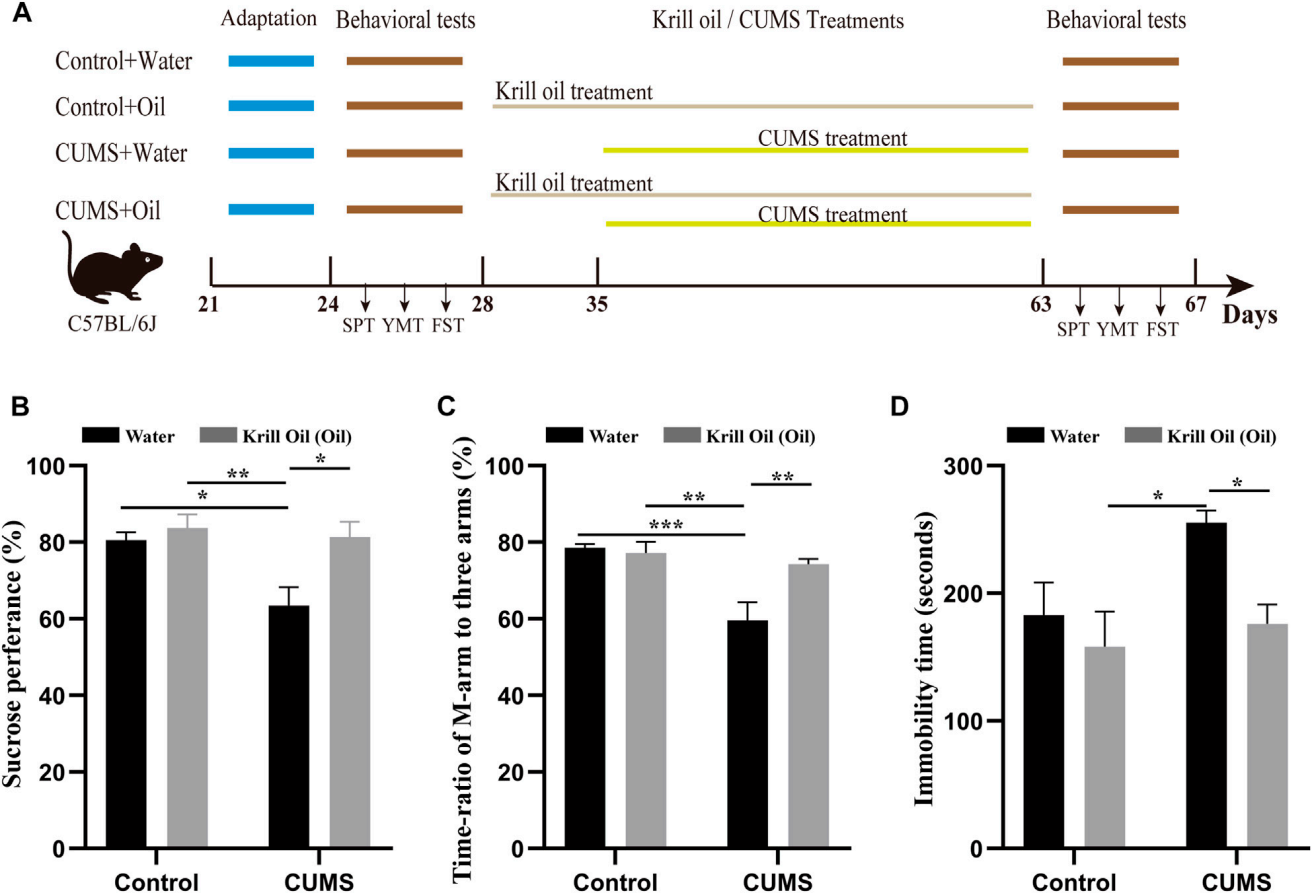
CUMS-induced depression-like behaviors were ameliorated by krill oil treatment. **(A)** Procedure for adaptation, behavioral tests, CUMS, and krill oil treatment. **(B)** Sucrose preference test values (%), C_W (*n* = 8), C_O (*n* = 8), MS_W (*n* = 8), and MS_O (*n* = 8). **(C)** Time ratios of M-arm to three arms (%), C_W (*n* = 8), C_O (*n* = 8), MS_W (*n* = 8), and MS_O (*n* = 8). **(D)** Immobile time of staying in the water cylinder (seconds), C_W (*n* = 8), C_O (*n* = 8), MS_W (*n* = 8), and MS_O (*n* = 8). Behavioral data were analyzed by two-way ANOVA and Tukey’s multiple comparisons test.

In the first week of the adaptation experiment, the mice’s body weight, forced swimming test (FST), sucrose preference test (SPT), and Y-maze test (YMT) were measured for the purpose of obtaining self-control. Then, the mice were divided into four groups (*n* = 8 per group), namely, Control + Water (C_W), Control + Oil (C_O), CUMS + Water (MS_W), and CUMS + Oil (MS_O). The scheme for CUMS treatment and krill oil supplementation was carried out, as shown in Figure 1A. Briefly, CUMS treatment included humid cages, social isolation, restrained spaces, inclined cages, empty cages, white noise, circadian rhythm disturbances, and strobe light (Strekalova et al., 2011; Hill et al., 2012), and were randomly carried out in a separate or combined manner for 4 weeks (Xu et al., 2016). From day 28, mice in C_O and MS_O groups received krill oil supplementation once a day at a dose of 200 mg (approximately 215 µL)/kg/d by intragastric administration, while mice in the C_W and MS_W groups were treated with the same volume of water. The composition of krill oil is shown in Table 1.

**TABLE 1:** List of krill oil components (350 mg).

Composition	Content
Cholesterol	<5 mg
Total n-3 PUFA	90 mg
EPA	50 mg
DHA	24 mg
Phospholipid	130 mg
Astaxanthin	17 μg

The paradigms for the CUMS included circadian disturbance, social isolation, strobe light, tilted cage, white noise, empty cage, restraint space, and damp sawdust cage (Supplementary Table S1). Except for social isolation, such treatments were administered to mice at random in separation or combination every day (Supplementary Table S2). These treatments’ durations and intervals were unpredictable to mice. CUMS was given to the mice for 4 weeks until they developed anhedonia and low self-esteem (Xu et al., 2016). Extreme stresses in a single pattern, such as learned social defeat, hopelessness, tail clamp, or electrical shock, were avoided in our study because they may induce fear memory-related outcomes that lead to anxiety, such as phobia to specific events and post-traumatic stressful disorder.

The SPT was performed with 1% sucrose water *vs.* water over 4 h, with the value provided as the ratio of consumed sucrose water to ingested sucrose water plus pure water. The YMT was studied by keeping a mouse in a specific arm and the other two arms for 3 min. A female mouse (dubbed M-arm) was attached to the end of this particular arm. The ratio of the stay time in the M-arm to that in three arms represented the M-arm stay time. The FST was performed by measuring the immobility time within 5 min in a water cylinder (10 cm diameter and 19 cm depth at 25°C ± 1°C). The mice were deprived of food and water for 3 h before the SPT to drive their desire to consume water. To reduce the effect of odor on the YMT, these arms were wiped with 70% ethanol and then water after each test. These tests were carried out in a calm room with no additional pressure, using the same circadian circle for all mice and observing their adaptation in the test environment.

### Metabolic profiling by liquid chromatography–mass spectrometry (LC-MS)

Mice were anesthetized with isoflurane, perfused with normal saline in intra-heart, and decapitated. The whole brain tissues were collected and thawed on ice, and the metabolites were extracted from 20 µL of each sample with 120 µL pre-cooled 50% methanol buffer. The mixture of metabolites was vortexed for 1 min and incubated for 10 min at room temperature, and then stored at −20°C overnight. The mixture was centrifuged for 20 min. Subsequently, the supernatant was transferred to 96-well plates. The samples were stored at −80°C prior to the LC-MS analysis. A pooled quality control (QC) sample was also prepared by combining 10 μL of each extraction mixture.

All samples were analyzed with a TripleTOF 5600 Plus high-resolution tandem mass spectrometer (SCIEX) with both positive and negative ion modes. Chromatographic separation was performed with an ultraperformance liquid chromatography (UPLC) system (SCIEX). An ACQUITY UPLC T3 column (100 mm × 2.1 mm, 1.8 µm, Waters) was used for the reversed-phase separation. For the separation of metabolites, the mobile phase consisted of solvent A (water and 0.1% formic acid) and solvent B (acetonitrile and 0.1% formic acid). The gradient elution conditions were as follows with a flow rate of 0.4 mL/min: 5% solvent B for 0–0.5 min; 5%–100% solvent B for 0.5–7 min; 100% solvent B for 7–8 min; 100%–5% solvent B for 8–8.1 min; and 5% solvent B for 8.1–10 min. The column temperature was maintained at 35°C.

XCMS software was used to analyze the obtained MS preprocessing data, including peak selection, peak grouping, retention time correction, second peak grouping, and isotope and admixture labeling. The KEGG and HMDB databases were used to annotate metabolites by matching the exact molecular weight of the sample (m/z) to the molecular weight in the database. The intensity of the peak data was further preprocessed by meta X. The Wilcoxon test was used to measure the difference in concentration between the two phenotypes. The use of FDR (Benjamini–Hochberg) was made to adjust *p*-values for multiple tests. The partial least squares regression method was used for discriminant analysis (PLS-DA) to distinguish among groups of different variables. The values of variables (VIP) that are important to the prediction were calculated, and VIP values greater than 1.0 were used as criteria.

### RNA purification from the brain tissues

Three mice were chosen at random from each group for RNA sequencing. The mice were anesthetized with isoflurane, perfused with normal saline in intra-heart, and decapitated. The whole brain tissues were collected and thawed on ice for RNA sequencing. Total RNA was extracted using the TRIzol reagent (Invitrogen, Carlsbad, CA, United States), followed by quality evaluation using the 2100 Bioanalyzer and the RNA 6000 Nano LabChip Kit (Agilent, Santa Clara, CA, United States).

### Library preparation and mRNA sequencing

Reverse transcription of the cleaved RNA fragments was performed by using the mRNA-Seq sample preparation kit (Illumina, San Diego, United States) to construct a cDNA library. Then, the collected samples were sequenced and analyzed on the Illumina HiSeq 4000 (LC Sciences, United States) machine and identified. The paired-end read of 300 bp (±50 bp) is the length we read. To ensure high-quality readings, we need to filter out low-quality readings according to the following three rules: 1) delete reads containing sequencing adaptors; 2) delete reads containing sequencing primers; and 3) delete nucleotides with a quality score lower than 10. For the analysis of RNA sequencing data results, the reads of samples were aligned to the UCSC (http://genome.ucsc.edu/) *Mus musculus* reference genome using the HISAT package. The mapped reads of each sample were assembled using the StringTie. Then, all transcriptomes from the samples were merged to reconstruct a comprehensive transcriptome using Perl scripts. After the final transcriptome was generated, StringTie and Ballgown were used to estimate the expression levels of all transcripts. StringTie was used to determine the expression level of mRNAs by calculating fragments per kilobase million (FPKM).

### Differentially expressed transcript analysis and data analysis

The differentially expressed genes (DEGs) were selected with |log2 (fold change)| >1 and statistical significance (*p*-value < 0.05) by the R package Ballgown.

### Statistical analyses

The initial processing raw data on mRNA expression profiles were performed by using the NOIseq software algorithm. The data on behavioral tests and gene analyses are presented as the mean ± SEM. Behavioral data were analyzed by two-way ANOVA and Tukey’s multiple comparisons test. The differential lipid components and differential genes between groups were statistically analyzed by unpaired Student’s *t*-test. *p* < 0.05 is considered statistically significant.

## Results

### CUMS-induced depression-like behaviors were ameliorated by krill oil in mice

To investigate the effect of treatment with krill oil on CUMS-induced depression-like behaviors, SPT, YMT, and FST were used to determine depression- and antidepression-like behaviors in mice after exposure to various stressors. As shown in Figure 1A, C57BL/6J male mice were divided into four groups, such as C_W, C_O, MS_W, and MS_O. Mice in the C_O and MS_O groups were treated with krill oil for 5 weeks, and mice in the MS_W and MS_O groups were treated with CUMS for 4 weeks. As shown in Figures 1B–D, in the MS_W group, the SPT values (83.26 ± 2.66 *vs.* 63.47 ± 4.79, *p* < 0.05, n = 8) (Figure 1B; Supplementary Tables S3, S4) and the ratios of stay time in M-arm to stay time in total arms (80.31 ± 2.68 *vs.* 59.59 ± 4.73, *p* < 0.01, *n* = 8) (Figure 1C; Supplementary Tables S5, S6) significantly decreased after 4-week treatments, and the values of FSTs immobile time were 255.3 ± 9.60 after treatment and 171.3 ± 33.79 before the CUMS treatment (*p* < 0.05, *n* = 8) (Figure 1D; Supplementary Tables S7, S8). These results suggested that mice exposed to CUMS over a long period of time exhibited depression-like behaviors. Furthermore, it is noteworthy that comparisons between MS_W and C_W, MS_W and C_O, and MS_W and MS_O showed significant differences in SPT, YMT, and FST, respectively (Figures 1B–D; Supplementary Tables S3–S8). Excitingly, we found that the SPT and YMT values significantly increased in the MS_O group compared with the MS_W group, and the values of FST’s immobile time significantly decreased in the MS_O group compared with the MS_W group (Figures 1B–D; Supplementary Tables S3–S8). These results suggested that CUMS-induced depression-like behaviors were ameliorated by krill oil treatment.

### Lipid composition was altered after CUMS treatment in the MS_W group

**FIGURE 2:**
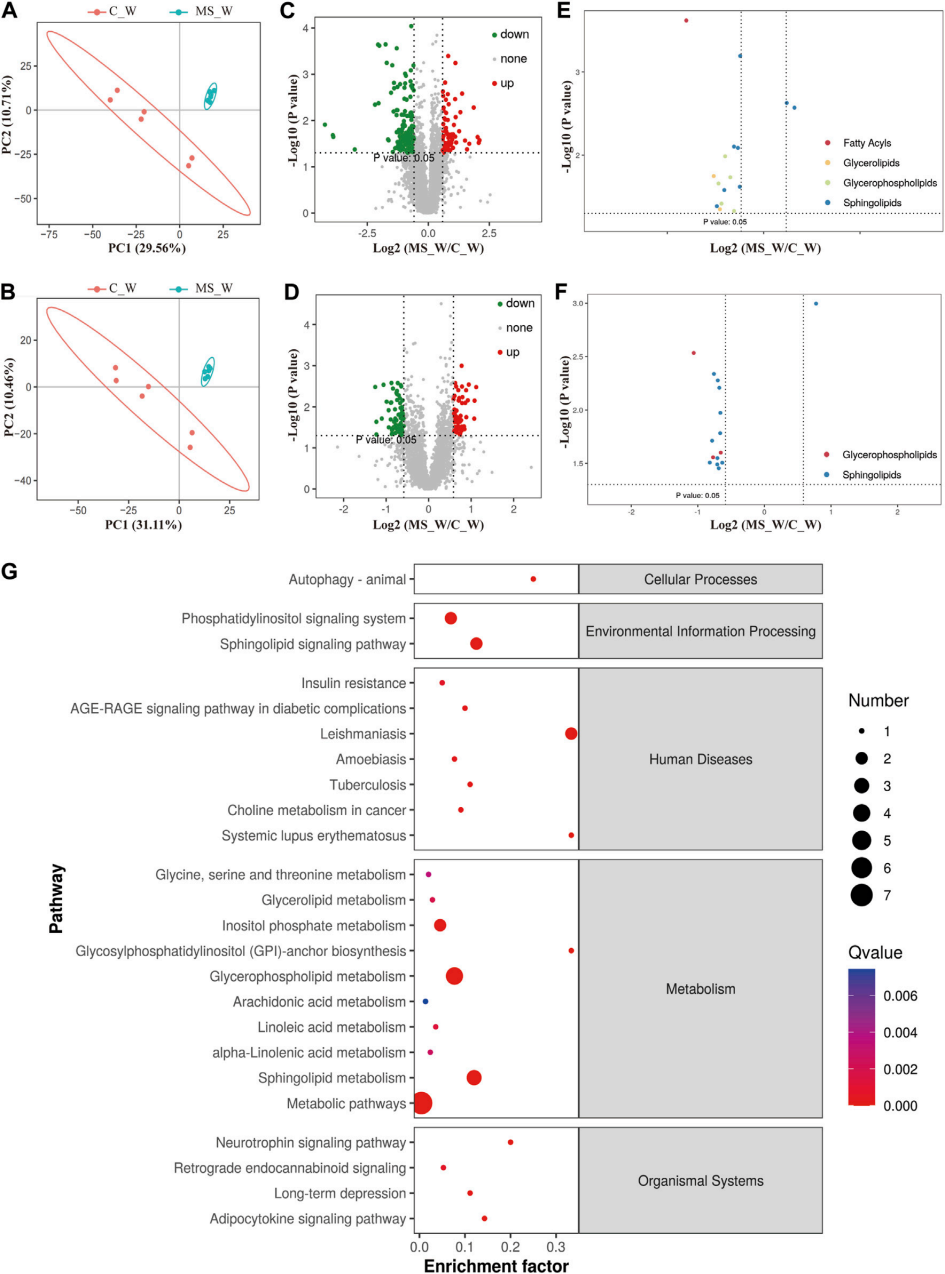
Lipid composition was altered after CUMS treatment. **(A,B)** PLS-DA score plots of samples prepared from C_W and MS_W mice under positive **(A)** and negative modes **(B)**. **(C,D)** Volcano plot analysis of the differential metabolites in C_W mice compared to MS_W mice under the positive **(C)** and negative modes **(D)**. **(E,F)** Volcano plot analysis of the differential metabolites cluster in C_W mice compared to MS_W mice under positive **(E)** and negative modes **(F)**. **(G)** Pathway enrichment analysis of potential biomarkers between the C_W and MS_W groups.

Although changes in lipid metabolism have been reported in some regions of the rat brain (Oliveira et al., 2016), the investigation of lipid metabolism in the whole brain is lacking. Here, we isolated the whole brain of mice in the C_W, C_O, MS_W, and MS_O groups after CUMS treatment, and analyzed the lipid metabolome using high-performance LC-MS (Supplementary Table S9). The pattern discriminant analysis of relevant data was carried out by partial least squares discriminant analysis (PLS-DA). As shown in Figures 2A, B, compared to the samples in the C_W group, samples in the MS_W group in both positive and negative modes were clustered tightly according to the PLS-DA scores, which indicated that significant alterations of endogenous metabolites were caused by CUMS treatment. As shown in Figure 2C, compared to the C_W group, 229 differential metabolites were observed in the MS_W group under the positive mode, of which 72 metabolites were upregulated and 157 metabolites were downregulated. In addition, there were 62 upregulated metabolites and 70 downregulated metabolites in the MS_W group under the negative mode when compared with the C_W group (Figure 2D). Furthermore, the volcano plot analysis of the differential metabolites in C_W mice compared to MS_W mice under the positive and negative modes also found that differential metabolites under the positive mode were mainly rich in fatty acyls, glycerolipids, glycerophospholipids, and sphingolipids, as well as glycerophospholipids and sphingolipids under the negative mode (Figures 2E, F). These results suggested that lipid composition was altered after CUMS treatment in mice.

### Identification of potential marker metabolites after CUMS treatments in the MS_W group

To further investigate the disturbance of lipid metabolism by CUMS, potential marker metabolites were identified based on the comparison of the spectrum of secondary fragment ions. As shown in Table 2, numerous metabolites were differentially regulated by CUMS treatment, such as acylcarnitine, ceramide [Non-hydroxy fatty acid sphingosine] (Cer[NS]), diacylglycerol (DG), sphingomyelin (SM), phosphatidylserine (PS), and plasmenyl-phosphatidylcholine (Plasmenyl-PC). Acylcarnitine is the intermediate metabolite produced during fatty acid β-oxidation (FAO) [19720082], and the acylcarnitine 17:3 level was significantly affected after CUMS treatment (∼4-fold decrease). In addition, most of these differential metabolites are ceramides including Cer[NS] 34:1, Cer[NS] 34:2, Cer[NS] 35:1, Cer[NS] 36:1, Cer[NS] 38:1, Cer[NS] 38:4, Cer[NS] 40:1, Cer[NS] 40:2, Cer[NS] 41:1, Cer[NS] 41:2, and Cer[NS] 42:2, and the amount of these ceramides consistently decreased after CUMS treatment. Interestingly, the levels of SMs (products of PS and ceramide) were elevated after CUMS treatment, accompanied by a decrease in PS 36:1, PS 36:2, PS 38:4, PS 40:4, and PS 40:6 levels. Furthermore, the levels of plasmenyl-PC 36:6 decreased in response to CUMS treatment, and docosahexaenoic acid (DHA)-related metabolites including PS (18:0/22:6) and PC (P-14:0/22:6) were lowered after CUMS treatment.

**TABLE 2:** List of differential metabolites for discrimination between the C_W and MS_W groups from brain analysis.

t_R_ (min)	Metabolite	Formula	Ionization mode	Measured m/z	Fold change	VIP score	*p*-value	FDR
2.12	Acylcarnitine 17:3	C_24_H_42_NO_4_	POS	408.31	0.25	3.41	0.0002	0.1508
4.15	Cer[NS] 34:2; Cer[NS](d18:2/16:0)	C_34_H_65_NO_3_	POS	536.50	0.57	1.74	0.0079	0.3152
4.47	Cer[NS] 34:1; Cer[NS](d18:1/16:0)	C_34_H_67_NO_3_	POS	538.52	0.62	1.54	0.02396	0.3548
4.81	Cer[NS] 36:1; Cer[NS](d18:1/18:0)	C_36_H_71_NO_3_	POS	566.55	0.49	1.97	0.026238	0.3548
4.83	Cer[NS] 38:4; Cer[NS](d20:3/18:1)	C_38_H_69_NO_3_	POS	588.53	0.62	1.66	0.0006	0.2012
5.11	Cer[NS] 38:1; Cer[NS](d20:1/18:0)	C_38_H_75_NO_3_	POS	594.58	0.58	1.62	0.00827	0.3189
5.19	Cer[NS] 42:2; Cer[NS](d18:1/24:1)	C_42_H_81_NO_3_	POS	648.63	0.38	2.00	0.0410	0.36099
4.77	DG 36:4; DG(16:0/20:4)	C_39_H_68_O_5_	POS	634.54	0.41	2.27	0.0178	0.3548
5.07	DG 38:4; DG(18:0/20:4)	C_41_H_72_O_5_	POS	662.57	0.46	2.05	0.0446	0.3651
4.59	SM 36:2; SM(d14:0/22:2)	C_41_H_81_N_2_O_6_P	POS	729.59	1.69	1.88	0.0024	0.2501
4.99	SM 36:1; SM(d14:0/22:1)	C_41_H_83_N_2_O_6_P	POS	731.60	1.98	1.95	0.0027	0.2501
5.11	PS 36:2; PS(18:1/18:1)	C_42_H_78_NO_10_P	POS	786.51	0.63	1.95	0.0219	0.3548
8.41	PS 38:4; PS(19:2/19:2)	C_44_H_78_NO_10_P	POS	812.54	0.43	1.97	0.0382	0.3580
4.32	PS 40:6; PS(18:0/22:6)	C_46_H_78_NO_10_P	POS	836.54	0.49	1.90	0.0103	0.3548
8.41	PS 40:4; PS(20:2/20:2)	C_46_H_82_NO_10_P	POS	840.57	0.53	1.69	0.0468	0.3678
4.81	Cer[NS] 36:1; Cer[NS](d18:1/18:0)	C_36_H_71_NO_3_	NEG	566.55	0.59	1.85	0.0046	0.2468
4.47	Cer[NS] 34:1; Cer[NS](d18:1/16:0)	C_34_H_67_NO_3_	NEG	538.52	0.63	1.60	0.0106	0.2669
4.61	Cer[NS] 35:1; Cer[NS](d17:1/18:0)	C_35_H_69_NO_3_	NEG	596.51	0.61	1.59	0.0324	0.2938
5.11	Cer[NS] 38:1; Cer[NS](d20:1/18:0)	C_38_H_75_NO_3_	NEG	638.56	0.63	1.70	0.0062	0.2502
5.13	Cer[NS] 40:2; Cer[NS](d18:1/22:1)	C_40_H_77_NO_3_	NEG	664.58	0.63	1.59	0.0165	0.2669
5.40	Cer[NS] 40:1; Cer[NS](d18:1/22:0)	C_40_H_79_NO_3_	NEG	666.60	0.62	1.56	0.0352	0.2987
5.54	Cer[NS] 41:1; Cer[NS](d18:1/23:0)	C_41_H_81_NO_3_	NEG	680.61	0.61	1.62	0.0283	0.2911
5.39	Cer[NS] 42:2; Cer[NS](d18:1/24:1)	C_42_H_81_NO_3_	NEG	648.63	0.43	1.73	0.0311	0.2913
5.39	Cer[NS] 42:1; Cer[NS](d18:1/24:0)	C_42_H_83_NO_3_	NEG	694.61	0.58	1.75	0.01944	0.2669
4.99	SM 36:1; SM(d18:1/18:0)	C_41_H_83_N_2_O_6_P	NEG	775.58	1.71	2.11	0.0010	0.2029
5.11	PS 36:2; PS(18:1/18:1)	C_42_H_78_NO_10_P	NEG	786.51	0.63	2.26	0.0029	0.2272
3.49	Plasmenyl-PC 36:6; PC(P-14:0/22:6)	C_44_H_76_NO_7_P	NEG	806.53	0.63	1.65	0.0251	0.2817
8.30	PS 40:4; PS(18:0/22:4)	C_46_H_82_NO_10_P	NEG	838.55	0.58	1.58	0.0278	0.2902

Note: tR, retention time; Cer[NS], ceramide [non-hydroxy fatty acid sphingosine]; DG, diacylglycerol; SM, sphingomyelin; PS, phosphatidylserine; Plasmenyl-PC, plasmenyl-phosphatidylcholine; VIP, variable importance projection.

For further investigation, pathway enrichment analysis was conducted based on the extracted differential metabolites. As shown in Figure 2G, the significantly altered potential marker metabolites were mainly distributed into three metabolic pathways, namely, glycerophospholipid metabolism, sphingolipid metabolism, and inositol phosphate metabolism pathway.

### Krill oil treatment ameliorating CUMS-induced lipid metabolism imbalance in mice

**FIGURE 3:**
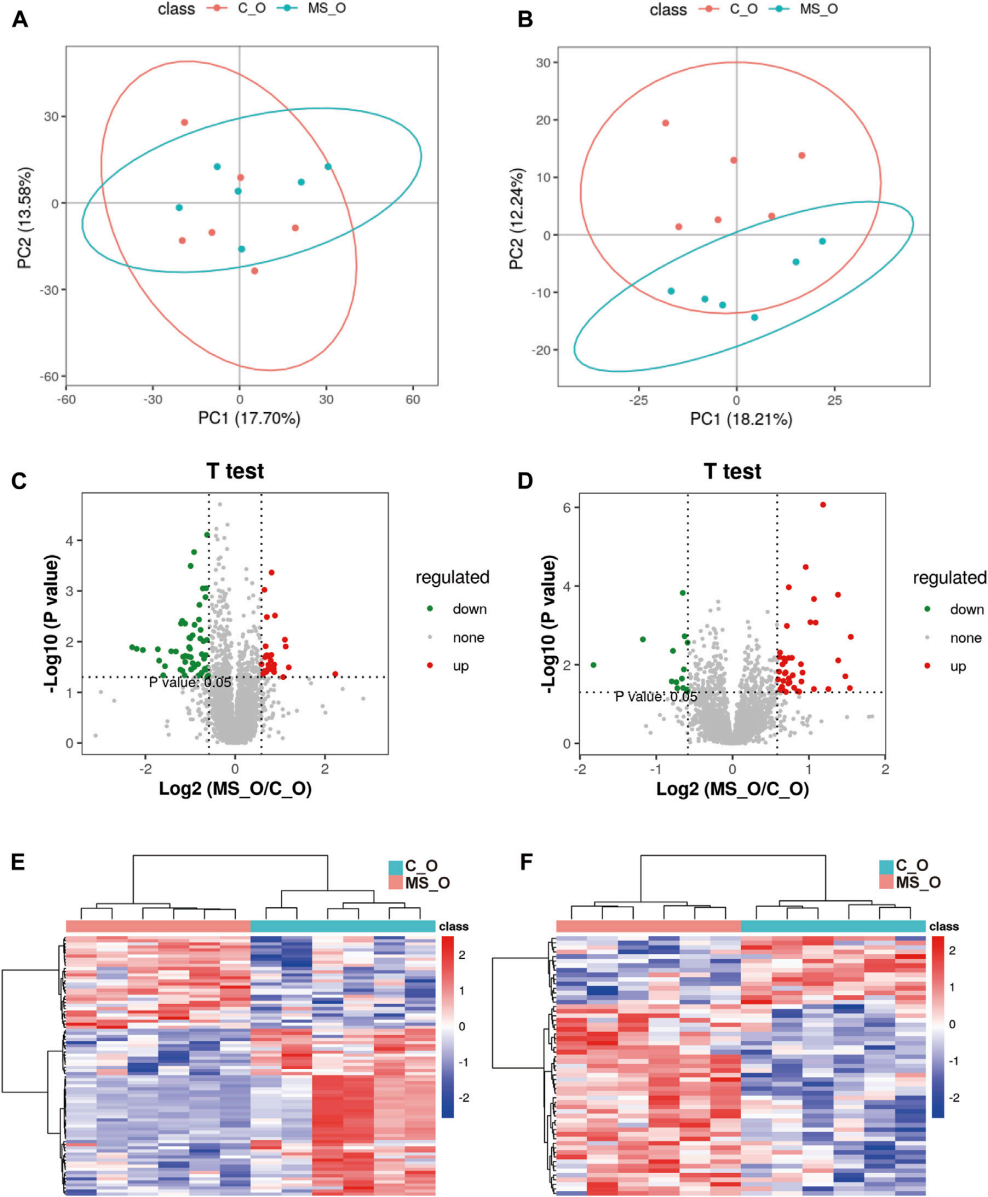
Krill oil supplement ameliorating CUMS-induced lipid metabolism imbalance in mice. **(A,B)** PLS-DA score plots of samples prepared from C_O and MS_O mice under positive **(A)** and negative modes **(B)**. **(C,D)** Volcano plot analysis of the differential metabolites in C_O mice compared to MS_O mice under positive **(C)** and negative modes **(D)**. **(E,F)** Heatmap of the level of differential metabolites in C_O mice compared to MS_O mice under positive **(E)** and negative modes **(F)**.

To further investigate whether krill oil ameliorates CUMS-induced lipid metabolism imbalance just as it ameliorates CUMS-induced depression-like behaviors, the lipid metabolism of the whole brain in the C_O and MS_O groups was analyzed (Supplementary Table S10). As shown in Figures 3A, B, compared to the samples in the C_O group, samples of the MS_O group in both positive and negative modes were not clustered tightly according to the PLS-DA scores, which indicated that CUMS treatment did not lead to significant alterations of endogenous metabolites in the presence of krill oil. In addition, compared with the C_O group, there were only 30 upregulated metabolites and 54 downregulated metabolites in the MS_O group under the positive mode, as well as 42 upregulated metabolites and 15 downregulated metabolites under the negative mode (Figures 3C–F). Furthermore, based on the comparison of the spectrum of secondary fragment ions, differential metabolites were identified between the C_O and MS_O groups. As shown in Table 3, only four differential metabolites, including glucosylceramide (GlcCer [NS]), triglyceride 52:3 (TG 52:3), triglyceride 54:3 (TG 52:3), and phosphatidylinositol 40:6 (PI 40:6), were identified. Interestingly, all the differential metabolites between the C_W and MS_W groups did not show significant changes in C_O *vs.* MS_O comparison. These results suggested that krill oil treatment ameliorated CUMS-induced lipid metabolism imbalances in mice.

**TABLE 3:** List of differential metabolites for discrimination between the C_O and MS_O groups from brain analysis.

t_R_ (min)	Metabolite	Formula	Ionization mode	Measured m/z	Fold change	VIP score	*p*-value	FDR
5.86	GlcCer[NS] 42:1; GlcCer[NS](d18:1/24:0)	C_48_H_93_NO_8_	POS	812.69	1.57	2.27	0.0401	0.5713
6.5	TG 52:3; TG(16:0/18:1/18:2)	C_55_H_100_O_6_	POS	874.78	0.51	3.24	0.03524	0.5606
6.63	TG 54:3; TG(18:1/18:1/18:1)	C_57_H_104_O_6_	POS	902.81	0.63	2.58	0.0360	0.5606
3.57	PI 40:6; PI(18:0/22:6)	C_49_H_83_O_13_P	NEG	909.53	0.66	2.60	0.0027	0.2387

Note: t_R_, retention time; GlcCer[NS], glucosylceramide [NS]; TG, triglyceride; PI, phosphatidylinositol; VIP, variable importance projection.

To further investigate the lipid metabolism difference between water and oil after CUMS treatment, the lipid metabolism of the whole brain in the MS_W and MS_O groups was analyzed (Supplementary Table S11). As shown in Table 4, three differential metabolites, namely, GlcCer [NS] 42:2 [GlcCer [NS](d18:1/24:1)], PC 40:2 [PC(18:1/22:1)], and LysoPE 20:1, were identified. Comparing to the samples in the MS_W group, the levels of GlcCer [NS] 42:2 and LysoPE 20:1 significantly increased in the MS_W group and the level of PC 40:2 decreased.

**TABLE 4:** List of differential metabolites for discrimination between the MS_W and MS_O groups from brain analysis.

t_R_ (min)	Metabolite	Formula	Ionization mode	Measured m/z	Fold change	VIP score	*p*-value	FDR
5.19	GlcCer[NS] 42:2; GlcCer[NS](d18:1/24:1)	C_48_H_91_NO_8_	POS	810.68	1.51	2.34	0.0338	0.8625
6.61	PC 40:2; PC(18:1/22:1)	C_48_H_92_NO_8_P	POS	842.66	0.59	2.40	0.0402	0.8654
1.81	LysoPE 20:1	C_25_H_50_NO_7_P	NEG	506.31	1.67	2.94	0.0314	0.6045

Note: t_R_, retention time; GlcCer[NS], glucosylceramide [NS]; PC, phosphatidylcholine; LysoPE, lyso-phosphatidylethanolamine; VIP, variable importance projection.

### Transcriptomic profiles of mouse brains under CUMS treatment with or without krill oil

**FIGURE 4:**
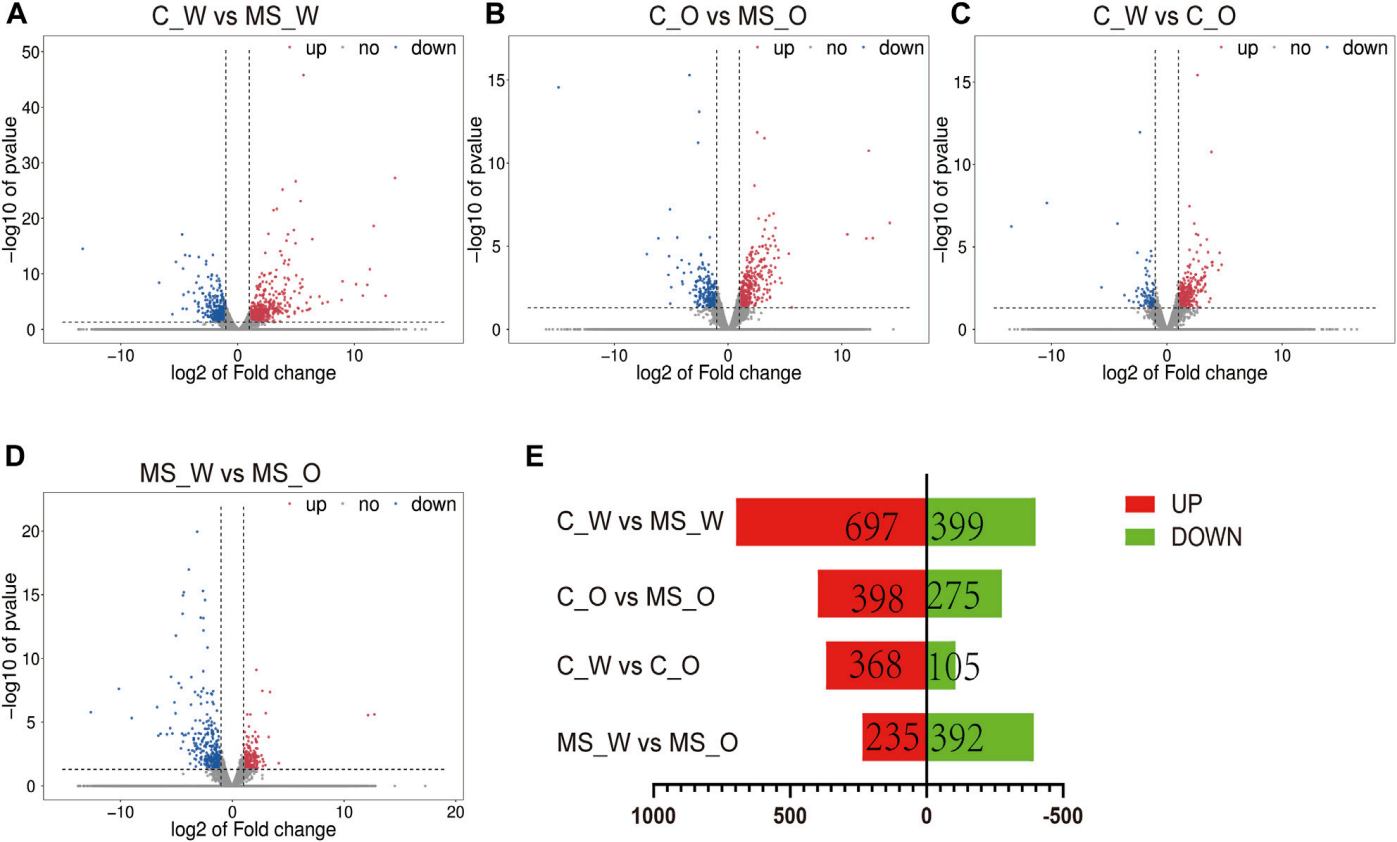
mRNA expression profiles of the whole brain in C_W, MS_W, C_O, and MS_O mice. **(A–D)** MA plot displays the DEG distribution between C_W and MS_W mice, C_O and MS_O mice, C_W and C_O mice, and MS_W and MS_O mice. **(E)** Bar graph shows the number of statistical DEGs.

To investigate the potential molecular mechanisms underlying krill oil treatment ameliorating CUMS-induced depression-like behaviors, transcriptomic analysis was carried out in the whole brain of mice from the C_W, MS_W, C_O, and MS_O groups. Overall qualities of RNA and the amount of reads mapped are shown in Supplementary Table S12. As shown in Figure 4 and Supplementary Table S13, compared to the C_W group, 1,096 DEGs were identified in the MS_W group, of which 697 DEGs were upregulated and 399 DEGs were downregulated. In addition, 673 DEGs were identified between the C_O and MS_O groups, of which 398 DEGs were upregulated and 275 DEGs were downregulated. These results indicated that the transcriptomic profiles of mouse brains were altered by CUMS treatment with or without krill oil.

**FIGURE 5:**
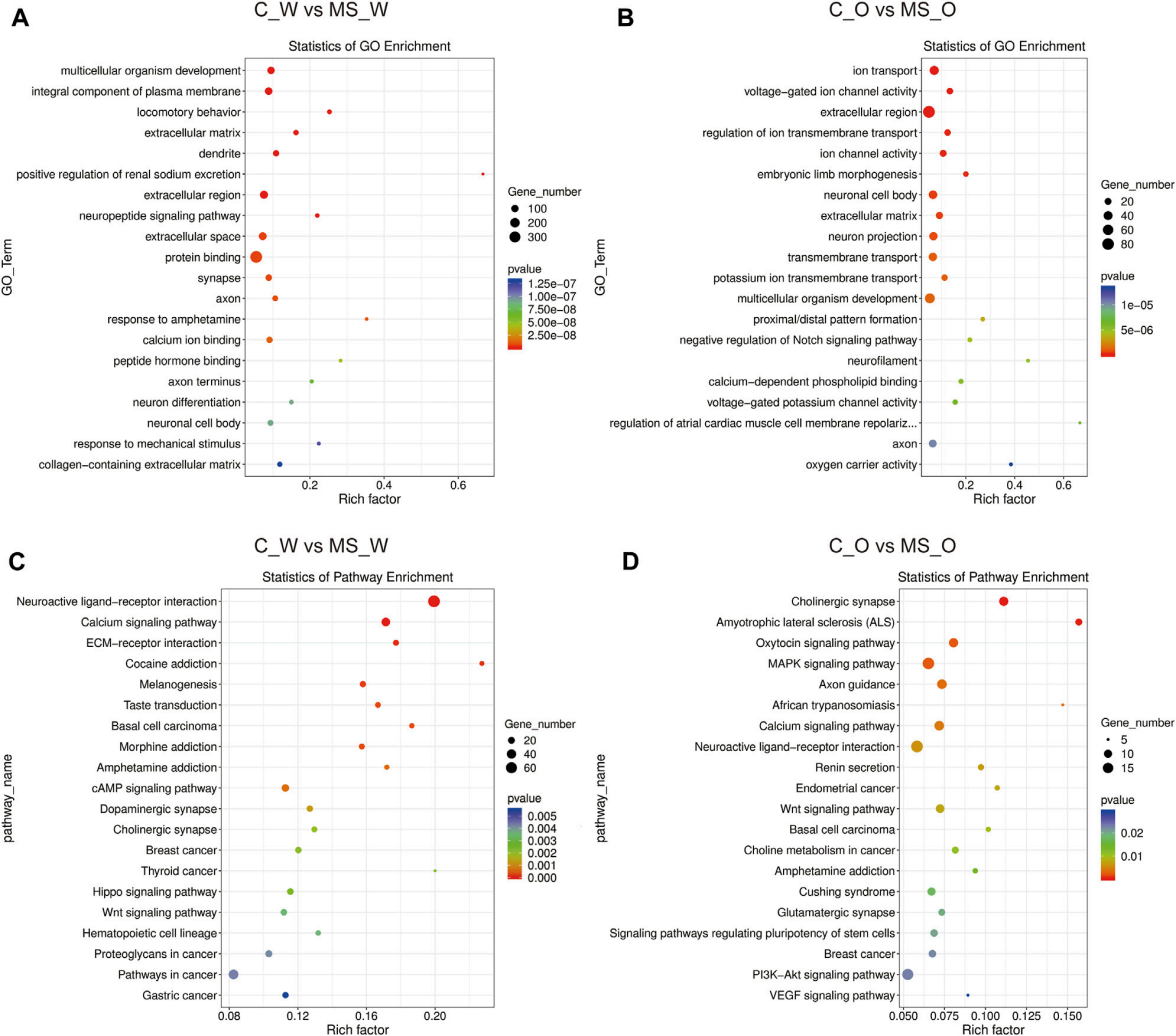
Functional analysis of DEGs under CUMS treatments with or without krill oil supplement. **(A)** Dot plot demonstrates DEG GO enrichment results in the C_W and MS_W comparison. **(B)** Dot plot demonstrates DEG GO enrichment results in the C_O and MS_O comparison. **(C)** Dot plot demonstrates DEG KEGG pathway enrichment results in the C_W and MS_W comparison; top 20 of pathway enrichment analyses are given. **(D)** Dot plot demonstrates DEG KEGG pathway enrichment results in C_O and MS_O comparison; top 20 of pathway enrichment analyses are given.

In order to obtain better insights into the molecular function of these DEGs, GO and Kyoto Encyclopedia of Genes and Genomes (KEGG) pathway enrichment analyses were performed in the C_W *vs.* MS_W (CUMS treatment without krill oil) comparison and the C_O *vs.* MS_O comparison (CUMS treatment with krill oil). In the C_W *vs.* MS_W comparison, the top 20 enriched GO terms included multicellular organism development (biological process, BP), integral component of plasma membrane (cellular component, CC), locomotory behavior (BP), extracellular matrix (CC), dendrite (CC), positive regulation of renal sodium excretion (BP), extracellular region (CC), neuropeptide signaling pathway (BP), extracellular space (CC), protein binding (molecular function, MF), synapse (CC), axon (CC), response to amphetamine (BP), calcium ion binding (MF), peptide hormone binding (MF), axon terminus (CC), neuron differentiation (BP), neuronal cell body (CC), response to mechanical stimulus (BP), and collagen-containing extracellular matrix (CC) (Figure 5A; Supplementary Table S14), which indicated that membrane structures appear to be affected after CUMS treatments. As shown in Figure 5B and Supplementary Table S15, in C_O *vs.* MS_O comparison, the top 20 enriched GO terms included ion transport (BP), voltage-gated ion channel activity (MF), extracellular region (CC), regulation of ion transmembrane transport (BP), ion channel activity (MF), embryonic limb morphogenesis (BP), neuronal cell body (CC), extracellular matrix (CC), neuron projection (CC), transmembrane transport (BP), potassium ion transmembrane transport (BP), multicellular organism development (BP), proximal/distal pattern formation (BP), negative regulation of the Notch signaling pathway (BP), neurofilament (CC), calcium-dependent phospholipid binding (MF), voltage-gated potassium channel activity (MF), regulation of atrial cardiac muscle cell membrane repolarization (BP), axon (CC), oxygen carrier activity (MF), and oxygen transport (BP).

KEGG pathway annotation results showed that DEGs in the C_W *vs.* MS_W comparison mainly participated in the neuroactive ligand–receptor interaction, calcium signaling pathway, ECM–receptor interaction, cocaine addiction, morphine addiction, amphetamine addiction, cAMP signaling pathway, dopaminergic synapse, and cholinergic synapse pathways (Figure 5C; Supplementary Table S16). In addition, DEGs in the C_O *vs.* MS_O comparison mainly participated in cholinergic synapse, amyotrophic lateral sclerosis (ALS), oxytocin signaling pathway, MAPK signaling pathway, axon guidance, African trypanosomiasis, calcium signaling pathway, neuroactive ligand–receptor interaction, renin secretion, and Wnt signaling pathway (Figure 5D; Supplementary Table S17).

**FIGURE 6:**
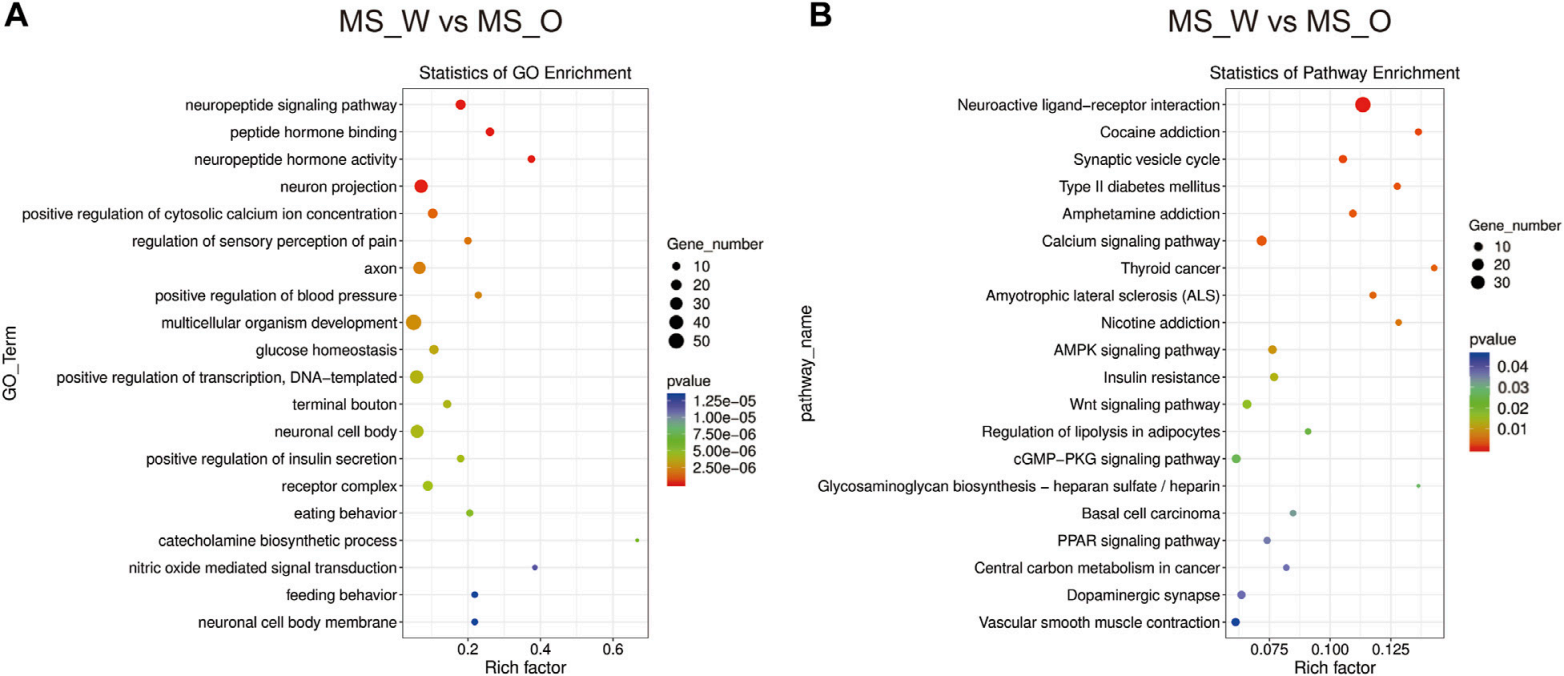
Functional analysis of DEGs in the MS_W and MS_O comparison. **(A)** Dot plot demonstrates DEG GO enrichment results in the MS_W and MS_O comparison. **(B)** Dot plot demonstrates DEG KEGG pathway enrichment results in the MS_W and MS_O comparison; top 20 of pathway enrichment analyses are given.

To further investigate the transcriptomic profile differences between water and oil after CUMS treatment, the transcriptomic analysis was carried out in the whole brain of mice from the MS_W and MS_O groups. As shown in Supplementary Table S18, compared to the MS_W group, 627 DEGs were identified in the MS_O group, of which 235 DEGs were upregulated and 392 DEGs were downregulated. In the MS_W *vs.* MS_O comparison (Figure 6A; Supplementary Table S19), the top 20 enriched GO terms included the neuropeptide signaling pathway (BP), peptide hormone binding (MF), neuropeptide hormone activity (MF), neuron projection (CC), positive regulation of the cytosolic calcium ion concentration (BP), regulation of sensory perception of pain (BP), axon (CC), positive regulation of blood pressure (BP), multicellular organism development (BP), glucose homeostasis (BP), positive regulation of transcription, DNA template (BP), terminal bouton (CC), neuronal cell body (CC), positive regulation of insulin secretion (BP), receptor complex (CC), eating behavior (BP), catecholamine biosynthetic process (BP), nitric oxide-mediated signal transduction (BP), feeding behavior (BP), and neuronal cell body membrane (CC). As shown in Figure 6B; Supplementary Table S20, DEGs in the MS_W *vs.* MS_O comparison mainly participated in the neuroactive ligand–receptor interaction, cocaine addiction, synaptic vesicle cycle, amphetamine addiction, calcium signaling pathway, amyotrophic lateral sclerosis (ALS), AMPK signaling pathway, Wnt signaling pathway, regulation of lipolysis in adipocytes, PPAR signaling pathway, and dopaminergic synapse.

## Discussion

MDD is a multi-factor disease and has many risk factors, including genetic and environmental factors. Fatty acids, an important nutrient in the diet, are thought to be associated with depression (Fernandes et al., 2017). In this study, we investigated the effect of krill oil on CUMS-induced depression-like behaviors by SPT, YMT, and FST, and found that the krill oil treatment prior to and during CUMS significantly ameliorated depression-like behaviors. In rats, the effects of krill oil on cognition and depression-like behavior were also evaluated, respectively, using the aversive light stimulus avoidance test (ALSAT) and FST, which indicated that active components (eicosapentaenoic acid, docosahexaenoic acid, and astaxanthin) in krill oil facilitate learning processes and provide antidepression-like effects (Wibrand et al., 2013). Using near-infrared spectroscopy and electroencephalography, Konagai et al. (2013) investigated the influence of ingestion of krill oil on cognitive function in elderly, found that krill oil was more effective than sardine oil in activating the cognitive function in the elderly, and concluded that these differences in the effects seem to be related to differences with regard to the incorporation of fatty acids into lipids. In addition, compared to conventional triacylglycerol-DHA, phospholipids-DHA and monoacylglycerol-DHA were efficient carriers of dietary DHA, and led to higher incorporations of DHA erythrocyte lipids (Destaillats et al., 2018). However, similar plasma and red blood cell levels of EPA + DHA were achieved across fish oil and krill oil products when matched for dose, EPA, and DHA concentrations in this 4-week study, indicating comparable oral bioavailability irrespective of formulation (Yurko-Mauro et al., 2015). In addition, the preventive and therapeutic effects of astaxanthin on depression-like behaviors have also been reported in high-fat diet and streptozotocin-treated rats, but the effective doses of astaxanthin in these studies were about 1000-fold higher than the doses provided in the work we report here (Ke et al., 2019). Likewise, although many studies have demonstrated the potential association between n-3 PUFAs and depression (Fernandes et al., 2017), the majority of n-3 PUFAs are incorporated into phosphatidylcholine in krill oil (Ahn et al., 2018; Song et al., 2022). Therefore, it is difficult to assign specific efficacies of n-3 PUFAs or astaxanthin to ameliorate depression-like behaviors and lipid alterations after the observed krill oil supplementation. Nonetheless, these observations suggest that higher doses along with higher-purity components of krill oil should be used in future studies to confirm the effects of krill oil components.

Prolonged exposure to stress can trigger impairments in the brain structure and function, which can lead to pathological processes to some diseases, such as Alzheimer’s disease and depression (Catania et al., 2009; Russell and Lightman, 2019). In this study, we carried out lipidomic analysis to study the impact of CUMS in the whole brain and found that lipid composition in the brain was altered after CUMS treatment. The metabolism of sphingolipids was disrupted with increased SM and decreased Cer levels (Figure 2; Table 2). This disruption has also been found in other studies, in which the levels of Cer in the prefrontal cortex (PFC) and hippocampus were found to increase after chronic stress, and SM decreased (Gulbins et al., 2013; Oliveira et al., 2016). However, no changes in the Cer levels were found in the amygdala and cerebellum (Oliveira et al., 2016). These differences may be caused by different brain areas or diverse fatty acid branches. Interestingly, compared with the C_O or MS_W groups, the level of GlcCer was significantly increased in the MS_W group. GlcCer is the simplest member and precursor of a fascinating class of membrane lipids, the glycosphingolipids, which are implicated in the pathogenesis of various diseases, including glycosphingolipidoses, peripheral neuropathies, and secretory diarrhea (Jeyakumar et al., 2002; van Meer et al., 2003; Zhang and Kiechle, 2004). The focus of our future attention will be on whether GlcCer plays a role in ameliorating depression-like behaviors and whether its role has brain area specificity.

Importantly, glycerophospholipid metabolism was also disrupted with the decreased PS and PC levels (Figure 2; Table 2), and the level of DHA incorporated in PS and PC was lowered in response to CUMS. This is consistent with what other studies have shown, in which DHA levels decreased in both the hippocampus and serum (Zadeh-Ardabili et al., 2019). DHA is a primary structural component of the brain and plays an extremely important role in the division of brain cells, nerve conduction, nerve synaptic plasticity, and nerve myelin production (Bazinet and Laye, 2014; Song et al., 2016). However, several randomized clinical trials have shown that n-3 PUFAs can improve the clinical depression scores in adults (Grosso et al., 2014; Mischoulon et al., 2015), which have been predominantly attributed to EPA (Grosso et al., 2014). Clinical studies have reported that EPA has antidepressant effects, especially when used in combination with DHA (Mocking et al., 2016; Mendoza et al., 2018). In our study, when supplemented with krill oil, CUMS did not lead to a decrease in DHA levels in the brain or cause severe depression-like behaviors. These studies indicated that DHA might be a predictive marker for depression. However, the relationship between DHA and symptoms of depression should be explored by a direct side-by-side comparison of pure EPA and pure DHA (Weiser et al., 2015).

The cause of depression is multifactorial, and the pathogenesis of depression is still the topic of scientific research (An et al., 2020; Song and Wang, 2021). In this study, CUMS led to depression-like behavior in mice, mRNA expression profiling was explored, and 1,096 DEGs were screened in the whole brain after CUMS treatment. Subsequent GO analysis found that these genes were mainly enriched in the integral components of the plasma membrane and the KEGG pathway in neuroactive ligand–receptor interaction, which was consistent with our previous transcriptome sequencing results in the NAc, amygdala, and VTA of depression-like mice (Si et al., 2018; Sun et al., 2018; Shen et al., 2019). Interestingly, the top 20 GO terms enrichment after supplementation krill oil during CUMS were not enriched in the integral component of the plasma membrane, and the KEGG pathways were also changed. These results suggested that krill oil supplementation not only changed lipid metabolism but also transcription. To explore the connection among transcription, lipid metabolism, and behaviors, we attempted to investigate the DEGs in the MS_W *vs.* MS_O comparison and uncovered some genes that may be implicated in the connection, such as *Gcnt1*, *Plekha2*, *Sgpp2*, and *B3galt1*. Their significance in the prevention and treatment of depression will need to be validated in the future by multidisciplinary research.

The major limitations to the present investigation were the use of water as a control rather than another neutral oil treatment. The association between oils and depression-like behaviors is complex, involving saturated and unsaturated fatty acids, n-3 unsaturated fatty acids, and n-6 multi-unsaturated fatty acids, as well as EPA and DHA. Therefore, various higher-purity components of krill oil should be used to study the relationship between brain function and fatty acid composition in the brain tissue. Taken together, we found that CUMS-induced depression-like behaviors were ameliorated by krill oil supplementation, with significant changes in mRNA and lipid composition in the whole brain. This study enhanced our understanding of the relationship between lipids and depression and provided new potential strategies for the prevention and treatment of depression.

## Data Availability

The data presented in the study are deposited in the Gene Expression Omnibus (GEO) repository, accession number GSE237760, https://www.ncbi.nlm.nih.gov/geo/query/acc.cgi?acc=GSE237760.
