# Supplementary material for: Krill oil treatment ameliorates lipid metabolism imbalance in chronic unpredicted mild stress-induced depression-like behavior in mice
Source: Front Cell Dev Biol. 2023 Jul 26;11:1180483. doi: 10.3389/fcell.2023.1180483 (PMC10411196; doi:10.3389/fcell.2023.1180483)
Supplement: Supplementary file 1 [file DataSheet1.zip › Supplementary Material/Table 1 + 2.DOCX]

**Table S1. CUMS stress**

| Stress | Definition |
| --- | --- |
| Circadian rhythm disturbance | Living with irregular illumination |
| Social isolation | Living in a cage alone |
| White noise | Audible hissing sound (70 dB) |
| Empty cage with water | Staying in a cage with water (100 ml) |
| Inclined cage | Staying in 45°-slanted cage |
| Empty cage | Staying in a cage without sawdust |
| Restraint space | Staying in a body-fitted container |
| Humid cage | Staying in wet sawdust-filled cage |
| Inclined cage with water | Staying in 45°-slanted cage with 50 ml water in its low end |
| Strobe light | Flashes at 2.5 Hz (500lux) |

**Table S2. CUMS operation process**

| Weeks | Days | Stress Combination and duration | Illumination |
| --- | --- | --- | --- |
| 1 | d1 | SL 21:00-10:00; EC 21:00-10:00 | 11:00-21:00 |
|  | d2 | RS 13:00-14:00 | 7:00-19:00 |
|  | d3 | HC 8:00-13:00; RS 15:30-18:30 | 11:00-19:00 |
|  | d4 | WN 11:00-15:00; HC 17:00-23:00 | 0:00-12:00 |
|  | d5 | RS 10:30-12:30; EC 14:00-21:00; SL 23:00-9:00 | 4:00-22:00 |
|  | d6 | EC 10:00-22:00; IC 22:00-14:00 | 12:00-18:00 |
|  | d7 | HC 10:00-16:00 | 19:00-5:00 |
| 2 | d1 | RS 15:00-16:00; WN 16:00-21:00 | 21:00-4:00 |
|  | d2 | RS 13:00-15:00; IC; SL 20:00-9:00 | 10:00-17:30 |
|  | d3 | RS 11:00-13:00; WN 18:30-10:30 | 8:00-20:00 |
|  | d4 | HC 9:30-18:00 | 22:00-3:00 |
|  | d5 | RS 9:00-10:00; SL 22:00-10:00 | 14:00-20:00 |
|  | d6 | ECW 10:00-20:00 | 6:00-17:00 |
|  | d7 | WN 9:00-14:00; EC 22:00-13:00 | 19:00-7:00 |
| 3 | d1 | EC; SL 21:00-11:00 | 3:00-13:00 |
|  | d2 | RS 13:00-15:00 | 14:00-22:00 |
|  | d3 | ECW 9:00-17:00; WN 20:00-2:00 | 0:00-8:00 |
|  | d4 | HC 13:00-21:30; SL 15:30-21:30 | 23:00-13:00 |
|  | d5 | RS 10:00-13:00; ICW; SL 21:00-9:00 |  |
|  | d6 | HC 9:30-21:30 | 5:00-12:00 |
|  | d7 | WN 10:30-16:30 | 17:00-23:00 |
| 4 | d1 | RS 12:00-15:00; EC; SL 21:00-11:00 | 6:00-21:00 |
|  | d2 | HC 11:30-21:30; SL 10:30-21:30 | 7:00-19:00 |
|  | d3 | RS 13:00-15:00; WN 9:30-16:30 | 11:00-19:00 |
|  | d4 | ECW 9:00-19:00; WN 11:00-20:00 | 0:00-12:00 |
|  | d5 | RS 10:00-13:00; ICW; SL 21:00-9:00 | 4:00-22:00 |
|  | d6 | WN 8:30-15:30 | 12:00-18:00 |
|  | d7 | ECW 11:00-20:00 | 17:00-23:00 |

Note: EC, empty cage; ECW, empty cage with water; IC, inclined cage; ICW, inclined cage with water; HC, humid cage; RS, restraint space; WN, white noise; SL, strobe light.
