# Supplementary material for: Krill oil treatment ameliorates lipid metabolism imbalance in chronic unpredicted mild stress-induced depression-like behavior in mice
Source: Front Cell Dev Biol. 2023 Jul 26;11:1180483. doi: 10.3389/fcell.2023.1180483 (PMC10411196; doi:10.3389/fcell.2023.1180483)
Supplement: Supplementary file 1 [file DataSheet1.zip › Supplementary Material/Table 12.DOCX]

**Table S12. High throughput sequencing mRNA data**

| **Sample Name** | **Raw Data** | **Valid Data** | **Valid Ratio**  **(reads)** | **Mapped reads** | **Unique Mapped reads** |
| --- | --- | --- | --- | --- | --- |
| **RC_O-1** | 49729982 | 48502980 | 97.53% | 96.20% | 78.35% |
| **RC_O-2** | 40875244 | 39952890 | 97.74% | 94.75% | 76.14% |
| **RC_O-3** | 36379166 | 35556570 | 97.74% | 94.55% | 75.11% |
| **RC_W-1** | 53466038 | 52182332 | 97.60% | 95.93% | 77.06% |
| **RC_W-2** | 51063766 | 49870450 | 97.66% | 95.09% | 76.96% |
| **RC_W-3** | 54263436 | 52964512 | 97.61% | 95.67% | 77.74% |
| **RMS_O-1** | 51669042 | 50407958 | 97.56% | 96.05% | 77.47% |
| **RMS_O-2** | 41516806 | 40519136 | 97.60% | 95.32% | 77.20% |
| **RMS_O-3** | 41194044 | 40176924 | 97.53% | 95.73% | 77.26% |
| **RMS_W-1** | 40535770 | 39519500 | 97.49% | 96.04% | 77.47% |
| **RMS_W-2** | 36455716 | 35552302 | 97.52% | 96.10% | 77.32% |
| **RMS_W-3** | 44610030 | 43570228 | 97.67% | 95.52% | 76.91% |
