# Supplementary material for: Krill oil treatment ameliorates lipid metabolism imbalance in chronic unpredicted mild stress-induced depression-like behavior in mice
Source: Front Cell Dev Biol. 2023 Jul 26;11:1180483. doi: 10.3389/fcell.2023.1180483 (PMC10411196; doi:10.3389/fcell.2023.1180483)
Supplement: Supplementary file 1 [file DataSheet1.zip › Supplementary Material/Table 3-8.DOCX]

**Table S3. Tukey’s multiple comparisons test of Sucrose preference test (SPT) in Figure 1.**

| **Multiple comparisons** | **Mean Difference.** | **Significant?** | **Summary** | **Adjusted P-value** |
| --- | --- | --- | --- | --- |
| Control+Water vs. CUMS+Water | 17.06 | Yes | * | 0.0227 |
| Control+Water vs. Control+Oil | -3.153 | No | ns | 0.9401 |
| Control+Water vs. CUMS+Oil | -0.8564 | No | ns | 0.9986 |
| CUMS+Water vs. Control+Oil | -20.21 | Yes | ** | 0.0041 |
| CUMS+Water vs. CUMS+Oil | -17.92 | Yes | * | 0.0120 |
| Control+Oil vs. CUMS+Oil | 2.297 | No | ns | 0.9728 |

**Table S4. Double-tailed analysis of Sucrose preference test (SPT) in Figure 1.**

| **ANOVA table** | **DF** | **F** | **Significant?** | **P-value** |
| --- | --- | --- | --- | --- |
| Interaction | 1 | 3.686 | No | 0.0655 |
| Oil Factor | 1 | 6.336 | Yes | 0.0181 |
| CUMS Factor | 1 | 7.507 | Yes | 0.0108 |

Note. two asterisks show p < 0.01, One asterisk show p < 0.05 in which two-way ANOVA was used for the comparisons among Control+Water, Control+Oil , CUMS+Water and CUMS+Oil groups.

**Table S5. Tukey’s multiple comparisons test of Y-maze test (YMT) in Figure 1.**

| **Multiple comparisons** | **Mean Difference.** | **Significant?** | **Summary** | **Adjusted P-value** |
| --- | --- | --- | --- | --- |
| Control+Water vs. CUMS+Water | 18.93 | Yes | *** | 0.0009 |
| Control+Water vs. Control+Oil | 1.324 | No | ns | 0.9898 |
| Control+Water vs. CUMS+Oil | 4.202 | No | ns | 0.7661 |
| CUMS+Water vs. Control+Oil | 17.61 | Yes | ** | 0.0013 |
| CUMS+Water vs. CUMS+Oil | -14.73 | Yes | ** | 0.0078 |
| Control+Oil vs. CUMS+Oil | 2.878 | No | ns | 0.9003 |

**Table S6. Double-tailed analysis of Y-maze test (YMT) in Figure 1.**

| **ANOVA table** | **DF** | **F** | **Significant?** | **P-value** |
| --- | --- | --- | --- | --- |
| Interaction | 1 | 7.140 | Yes | 0.0126 |
| Oil Factor | 1 | 13.18 | Yes | 0.0012 |
| CUMS Factor | 1 | 4.979 | Yes | 0.0342 |

Note. Three asterisks show p < 0.001, two asterisks show p < 0.01 in which two-way ANOVA was used for the comparisons among Control+Water, Control+Oil , CUMS+Water and CUMS+Oil groups.

**Table S7.** **Tukey’s multiple comparisons test of Forced swimming test (FST) in Figure 1.**

| **Multiple comparisons** | **Mean Difference.** | **Significant?** | **Summary** | **Adjusted P-value** |
| --- | --- | --- | --- | --- |
| Control+Water vs. CUMS+Water | -72.39 | No | ns | 0.0918 |
| Control+Water vs. Control+Oil | 24.73 | No | ns | 0.8367 |
| Control+Water vs. CUMS+Oil | 6.875 | No | ns | 0.9955 |
| CUMS+Water vs. Control+Oil | -97.13 | Yes | * | 0.0107 |
| CUMS+Water vs. CUMS+Oil | 79.25 | Yes | * | 0.0461 |
| Control+Oil vs. CUMS+Oil | -17.88 | No | ns | 0.9229 |

**Table S8.** **Double-tailed analysis of Forced swimming test (FST) in Figure 1.**

| **ANOVA table** | **DF** | **F** | **Significant?** | **P-value** |
| --- | --- | --- | --- | --- |
| Interaction | 1 | 1.758 | No | 0.1960 |
| Oil Factor | 1 | 4.820 | Yes | 0.0369 |
| CUMS Factor | 1 | 6.395 | Yes | 0.0176 |

Note. One asterisk show p < 0.05 in which two-way ANOVA was used for the comparisons among Control+Water, Control+Oil , CUMS+Water and CUMS+Oil groups.
